
JOURNAL INFORMATION
==============================
NLM Title Abbreviation: Med Chir Rev
NLM Journal ID: 0034617

The Medico-Chirurgical Review

Publisher: Biomedical Journal Digitization Project

ARTICLE INFORMATION
==============================
PMCID: PMC4966189
PMID: 29917611
Article version: 1
Subjects: Articles

Hospital Practice

Print publication date: 1828 Jul 1
Volume: 9
Issue: 17
First page: 265
Last page: 273
1828 Jun 21
License: This work is free of known copyright restrictions. For more information, please see PMC Back Issue Digitization.
License URL: https://creativecommons.org/publicdomain/mark/1.0/

==============================

96. MEDICAL STATISTICS.

Modern records and researches would seem to hold out some prospect of immortality to man, even in this world. At all events, we may fairly hope that the "march of intellect" will slacken the pace of TIME, or so shorten the length of his scythe, that he will not mow down one half the number which he has been wont to do. Whether this change may be attended by a proportionate increase of happiness among mankind, is another question, and one which we cannot pretend to solve. Dr. Bisset Hawkins will certainly contribute to lower the premiums on life insurance, in this country, though peradventure his *assurances* may ruin some of the numerous companies which must start on the prospect of the increasing longevity of mankind. It is not our intention, however, to enter on the laborious researches of the learned Doctor, as detailed in his recent Lectures, at the College of Physicians. We shall, in this place, glance at some medical statistics which have been laid before the Royal Academy of Medicine, by Dr. Vilermé, respecting the connexion of births and deaths with rigorous and mild seasons—scarcity of provisions—healthy or unhealthy places of residence—political events—manners—industry—in a word, the entire state of civilization. The author comes to the following conclusions, as far as PARIS is concerned. The actual annual mortality of its inhabitants is 1 in 32. In the 17th century it was 1 in 25 or 26. In the 14th century it was 1 in 16 or 17. Formerly the deaths in Paris considerably exceeded the births:—now, the *latter* predominate. There is one birth annually among every collection of 29 or 30 inhabitants. The maximum of mortality, at present, is in the Spring:—formerly, it was in the Autumn, especially in the months of August and September. There are more male than female still-born children—and there is greater mortality, during the first three months (after birth) among the former than the latter. "The month of June shews the maximum of conceptions and the maximum of births." It is difficult to account for this fact; for if most conceptions take place in June, most births should occur in March or April. Indeed, we imagine that *maximum* in the latter part of the sentence should be *minimum;* for immediately afterwards we have the following statement.—"Le mois de Mars et d'Avril sont ceux qui presentent le plus grand nombre de naissances."

The opulent and the indigent classes of society present great differences in respect to relative mortality. Thus, in the richer quarters of the capital, there is 1 annual birth to 41 inhabitants. In the poorer quarters there is 1 birth to 29 or 30 of the population. In this calculation, the children of the poor, born in hospitals, are not taken into account. These would, of course, greatly increase the number of births among them. Nevertheless, it is found, that among the opulent classes of society, there are more children living under the age of 5 years, than among the indigent:—the inevitable conclusion is, that, although the poor people beget more children than the rich, they do not preserve so many.

Of 100 children abandoned (this must allude to asyla for the illegitimate) 60 perish within the first year. This ratio of mortality, however, among "*les enfans trouvés*," is annually diminishing. Scarcity or famine greatly diminishes the number of births. The fecundity attendant on marriages has been regularly diminishing during the last hundred years. The reasons assigned by the author for this decrement, we shall give in his own words. "C'est aux froids calculs de la fortune, c'est aux prévoyances extrémes suscitées par le goût du luxe ou par l'amour de l'aisance qu'il faut sans doute en attribuer la cause."

The talented author passes in review, year by year, since 1680, the great moral, political, and physical events which have occurred in Paris, and their influence on the population. The general conclusion is:—"that, each time the people suffer, whatever may be the cause, the number of deaths is increased—the number of births is diminished, and the mean duration of life is curtailed. On the other hand, whenever the people are happy, the reverse of the above obtains."

Not being acquainted with all the data on which Dr. Hawkins founds his cheering prospects of increased health and longevity, we shall not attempt to invalidate his positions; but we shall take the liberty of commenting on a few points, which happen to come within the scope of our own observation. The Doctor appears to us, to attribute a great deal more to medical art in the preservation of life, than he is justified in doing. Thus, he says, that in fevers, if left to themselves, there would probably be about one death in two; while treated according to modern scientific principles, *six out of seven*, or even *eleven out of twelve* will be saved! We are extremely sceptical as to the soundness of this position—nay, we verily believe, that taking all circumstances into account, nearly as many fever patients would recover on *whey*, as on the most costly and polypharmic treatment that modern medicine could devise. The Doctor evidently looks to the favourable side of the question on all occasions. We shall only be able to glance at one or two illustrations which he brings forward. "To mark the improvement of health in our navy," says he, "we may compare the fate of Commodore Anson's crew, with a ship placed in *similar circumstances* about fifty years afterwards. Anson passed 143 days at sea, without touching at any place of refreshment. On his arrival at Juan Fernandez, half of his companions alone survived; and of the remaining 200, only 8 were efficient. But, in 1794, the Suffolk, a 74 gun-ship, during 162 days, had no communication with land, and arrived in India without the loss of even one man." But surely Dr. Hawkins is not in earnest when he makes the Anson, struggling round Cape Horn, against adverse winds, and among dreary, barren, and dangerous rocks, where hope was extinguished and human strength exhausted—to be *similarly circumstanced* with the Suffolk, sailing on velvet, before the balmy trade winds to the land of pagodas, at the beginning of the war, in which every common sailor expected to make a fortune! Perhaps, in the whole annals of navigation, from the Periplus of Hanno, down to Parry's "*Noctes Atticæ*," among the ice-bergs, so remarkable a contrast could not be found as that presented by the *circumstances*, moral and physical, in which the crews of the Anson and Suffolk were placed. But, let us come down nine or ten years later, and see what happened. A frigate, just fitted out with a wretched heterogeneous crew, was dispatched, without an hour's preparation, for the East Indies, in order to give notice of the new war which broke out in 1803. That ship went to the East Indies, without losing a man, without presenting a single symptom of scurvy, though the crew lived on salt-junk during the whole of the voyage. Here, says Dr. H. is a fine illustration of my position. Softly kind Sir! Two other ships were fitted out for the East India station, immediately after the one alluded to; but they were stored with lime-juice, and every thing that could be supposed contributing to health on a long voyage. They sailed, and they arrived at Madras complete lazarettos, the crews being eaten up with scurvy and scorbutic ulcers! In short, they were pretty much in the condition of the Anson's crew. How is this to be accounted for? Readily enough. The first ship that sailed was peculiarly fortunate. She took prizes in every parallel of Latitude, and the crew were elated beyond measure at the success already obtained, and the prospect of its continuance. The other two ships sailed with similar hopes; but they were cruelly disappointed. Not a Frenchman ever crossed their track—not a shilling of prize-money did they make! The consequence of these mental depressions were corporeal diseases.

Dr. B. seems to pity the Roman Legions, who had only one medical officer to three or four thousand men. They were still worse off, we should imagine, at the siege of Troy. But when we reflect on the powers of dame Nature, and the hardy constitutions of those times, we doubt whether the paucity of Doctors made much difference in the general average of mortality. In ordinary circumstances—in accidents—and in inflammatory diseases, medicine is very powerful; but when the epidemic influence has once gone abroad in the atmosphere, small is the share which the most consummate medical skill has in checking the mortality. The plains of Bengal, the islands of the West, the swamps of Walcheren—nay, the very rock of Gibraltar, which is quoted by Dr. Hawkins, afford melancholy proofs of the position here mentioned. The increase of longevity, then, we are inclined to attribute to moral and physical causes which are little under the control of medical art. Indeed, we are forced to agree with M. Vilermé, that—when people are *happy* they will be healthy and long-lived, and *vice versâ*.

---

### 97. Extraordinary Instances of Suppression and Retention of Urine.

In Hufeland's Journal for August, 1827, there is an extraordinary case related of a young lad, who made no water for seven weeks, and who suffered little or no inconvenience from this extraordinary *suppression*. There was no vicarious secretion in this case. Catheters were introduced into the bladder, but no urine could be found. Dr. Racum (of Riga) after failing with all other medicines, restored the urinary secretion by a mixture of oil of amber, venice turpentine, and balsam of capivi.

In another German Journal, there is a case of *retention* quite as extraordinary. The patient was a young lad of 13 years of age, who experienced a severe paroxysm of fever, in October, 1822, followed by a miliary eruption, which was repelled by exposure to cold. Another paroxysm ensued, and great debility was the result. On the 20th November, he was seized with violent pain in the rectum, which was greatly aggravated by attempts to pass any fæcal matters. In the beginning of January following, the constipation still continuing, the urinary secretion became extremely scanty, and at length ceased for fifteen days. This cessation, and for a similar space of time recurred twice afterwards. In March, 1823, the urinary discharge ceased again, and none was passed for *six months* afterwards! In July, the boy was found to be extremely emaciated, the bowels being obstinately constipated, and there being periodical pains in the loins, which were so severe as to throw him into convulsions. Nothing unusual could be discovered about the urinary passage, or rectum. The abdomen, however, was greatly distended, but not apparently with fluid, as it emitted a hollow sound when struck. On exploring the rectum there was felt, on each side, an elastic tumour, the nature of which could not be ascertained. Dr. Berres introduced a very fine catheter, the urethra being extremely contracted, but could not draw off any urine. Medicines only exasperated his sufferings. He was taken by his father to Pest, where he was examined by several physicians and surgeons. While there, the water commenced flowing in large quantities—the abdomen shrunk in proportion—and the boy got well.

We cannot but conclude, that this was retention, rather than suppression of urine, but it is difficult to believe that such retention could last so long without destroying life. There does not, however, appear to be any reason for deception, either on the part of patient or practitioner, in this case.

---

### 98. Simultaneous Extra, and Intra-Uterine Fœtation.

The following case is communicated by Dr. Detwiller, to the Philadelphia Journal of Medicine and Surgery.

Mrs. Kremer, aged 33 years, became affected, a few months after marriage, with pain in the left side of the abdomen, low down, accompanied by gradual enlargement of the abdomen and mammæ—nausea—vomiting;—but regularity of the menses, for nearly two years, when they ceased. On the 6th January, 1825, Dr. D. found the patient labouring under violent pain in the abdomen, urgent desire to make water, vomiting, constipation, hard full pulse. The remedies used need not be specified. On the 23rd of April, she was again examined, and Dr. D. found the left ovarium much enlarged, hard, but without tenderness. The distended uterus could also be felt, independent of the enlarged ovarium. Pregnancy proceeded, and the woman was delivered of a living, healthy child, on the 22nd of October. On the 3rd day after delivery, the ovarian tumour became painful—inflammation came on, with corresponding febrile symptoms, and the patient died on the eleventh day after delivery.

*Dissection.* Eight pints of serous fluid were found in the abdomen. The left ovarium was found greatly enlarged, extending into the right hypogastrium, and covering the uterus. In the right side of this tumour was an opening, through which, a fluid, similar to that observed in the abdomen, issued on pressure. There were gangrenous spots on the intestines, as well as on the uterus. The left fallopian tube was pervious, but the corresponding ovarium was dropsical, and on laying it open, the crowns of four molar teeth and two incisors came into view—the former attached to an irregularly shaped piece of bone. Throughout this cavity were interspersed a number of whitish hairs, some of them in tufts. A number of bones, of various sizes, were found imbedded in the more solid parts of the ovarian tumour, but none of them presented the shape of human bones.

In the Philosophical Transactions, there is the case, of a woman who carried an extra-uterine fœtus 18 years, in which time she was safely delivered of a living child. Two other cases, of nearly a similar kind, are also related in the same collection.

An interesting case of extra-uterine fœtation has recently been recorded by Mr. Tilt, (of ———, Gray's-Inn Lane,) in the May No. of the Medical and Physical Journal. A woman, aged 37, was exposed to much fatigue, in the third month of her fifth pregnancy, when she felt something give way internally, followed by a discharge of blood from the vagina, vomiting of the same, anorexia, tenderness of abdomen, retention of urine, and obstinate constipation. These symptoms required purgatives, and the use of the catheter for some time. It was nearly five months after the commencement of these symptoms, when Mr. Tilt saw the patient, she being then emaciated, and her strength greatly exhausted by frequent vomiting, a fetid discharge from the vagina, and incessant abdominal pain. She was apparently as large as in the seventh or eighth month of utero-gestation. Little doubt was entertained as to the real state of the case, and the patient soon afterwards died.

*Dissection.* Dr. Davies, Dr. Dill, and Mr. Bell, assisted Mr. Tilt in examining the body. The skin was jaundiced—the extremities slightly œdematous. On opening the abdomen, a large quantity of bloody fluid escaped. The peritoneum was highly inflamed and thickened—small intestines pushed up against the diaphragm. Between the uterus (rather larger than in the unimpregnated state) and the intestines, lay the fœtus, involved in its membranes, the placenta being exterior to, and betwixt them and the arch of the colon. The membranes being opened, the fœtus appeared to be rather smaller than one at full term. The appearances on the surface led them to think, that the child had been dead some time. The chorion and amnion were perfectly natural, and exterior to these lay the sac, or ventral uterus, within which, the fœtal mass, with the exception of the placenta, lay. The walls of this sac were three lines in thickness, and perfectly smooth within. The placenta was of enormous size, and very extensively attached, weighing five pounds. It adhered with great tenacity to the sac. It communicated freely with the left mesenteric and emulgent vessels, and its supply of blood must have been very liberal. The uterus was large, but presented no appearance of having been ruptured. About the middle of the left fallopian tube, its coats were ulcerated for about an inch and a half. Mr. Tilt has made a great many ingenious observations on the above case, for which we must refer to the original paper.

---

### 99. DR. LAMBE ON THAMES AND RIVER WATER.*

> For not to know at large of things remote
> From use, obscure and subtle, but to know
> That which before us lies in daily life,
> Is the prime wisdom. *Milton.*

There being now but one opinion as to the impurity and insalubrity of the water with which a considerable portion of the metropolis is supplied, the minds of men are naturally led to inquire into the remedy. Dr. Lambe, who has long turned his attention to the properties of water and its extraneous admixtures, now presents us with a pamphlet, in which he has taken great pains to examine the water of the Thames, not only about London, but

---

* An Investigation of the Properties of Thames Water. By William Lambe, M.D. 8vo. sewed, p. 65. 2s. 6d. 1828.

higher up, and beyond the reach of the tide—consequently where the Thames is in the same condition as other rivers. The inquiry is therefore interesting, as affecting not merely the water of the Thames, but of the New River, and indeed of every river. The following "General conclusion" we shall extract.

"The result of the examination of the Thames water at London, has proved that every product of it which can be obtained and exhibited in a distinct form, is tainted, or to speak more truly, is wholly composed of the *exuviæ* of animal and vegetable matter. We have extracted bodies which can be traced to no other origin out of the soluble saline matter; out of the insoluble residuum; out of the hardly soluble sulphate of lime; finally, out of that insoluble matter separated by simple boiling, and improperly called carbonate of lime. We have shewn that the insoluble residuum itself is partly at least, if not totally, volatile at the low degree of heat of boiling water, a property which distinguishes it completely from all common earthy matter, and entitles it to be ranked among organic bodies. We have extracted from it oily substances, which can be traced to no other source than to the accumulation of organic remains. Finally, we have extracted abundantly the constant residuum of organic bodies, a charcoally matter, by the simple process of solution in acid (under peculiar circumstances) which I conceive could never happen but with a water deeply and incurably tainted with heterogeneous substances, deposited from the filth with which it is incessantly mingled. No man, therefore, I should think, who calls himself a chemist, or who aspires to the more exalted title of a philosopher, can have the hardihood to deny that this water is loaded with animal and vegetable putridity, in as high a degree as it is possible, in any which the human organs can sustain. And all those who, thinking with me, consider such matters to be deleterious and destructive of animal life, must join in condemning it as unfit to be applied to any dietetic purpose whatever.

"If we trace the river higher up to the point where the tide nearly ceases to be observed, strong presumption has been afforded that the same mischief predominates, but in a somewhat mitigated form. Circumstances which have been already detailed have prevented my acquiring the evidence on this point which it was intended and desired. But I entertain no doubt that it will be established, that the pollutions of the river received at the metropolis are carried upwards as far as the influence of the tide extends."

The water of the Thames at Windsor, does not appear to have been so minutely examined as the London water; but our author seems to conclude that both it and all other rivers "must become loaded with whatever soluble matter an abundant putrefaction can supply, wherever the said river or rivers run through a rich and fertile country with a dense population." While we cannot deny that a certain proportion of foreign and corrupt matters must necessarily exist in all streams that run through fertile soils, and near the habitations of men and animals, yet we apprehend that we can only balance between the greater and the lesser evil—and that we must and ought to be contented, if we can procure our general supply of water from such a running stream as does not pass through any large town. Dr. Lambe observes that there are only four modes of purifying foul water—chemical agents—boiling—filtration—distillation. The two first are out of the question; but, "filtration through charcoal frees water from putrid and perhaps from putrescent vapour, and so far must be preferable to every other mode of filtration."*

But distillation is considered by Dr. Lambe as the only process which can render water perfectly pure and perfectly wholesome. Dr. L. has brought forward a considerable number of cases illustrating the beneficial effects of distilled water on cancer and other local affections; and the Doctor pretty openly accuses Mr. Abernethy of stealing from him the facts

---

* The ingenious apparatus invented by Messrs. Robins and Co. appears to us to be the best filtering machine yet put to use. It fits into the reservoir, and therefore all the water used by the family for culinary purposes, must pass through the filter, which renders the water (and we have now used the apparatus for some months) as clear as crystal, and without taste or smell. We strongly recommend this filter to every family.

and information, "which have formed the ground-work of his present opinions." Dr. L. does not propose any scheme by which distilled water might be afforded, on a large scale, to the metropolis; the process, therefore, of distillation can only apply to that portion of water which is drunk by each individual, and this must be purchased. The present price of distilled water is 4d. per gallon; but Dr. L. thinks it might be procured at one fourth of that sum. The conclusion to which most men, we think, will come, is, that water should be supplied from a running stream, as the New River is now supplied; and that we must put up with such impurities as the filter cannot remove from it. We shall never cease, however, to hold up our hands against the Thames water taken up nearer than Windsor.

### 100. Consolation for English Medical Men on the Continent.

The Clinique des Hôpitaux, in one of its numbers for October last, copies one of those "scenes," or theatrical representations, [misrepresentations,] for which a weekly journal, in this country, is so famous; namely, certain dialogues, said, by the veracious journal, to have occurred between Sir A. Carlisle, Mr. White, Mr. Harding, and a little boy who was undergoing the operation of lithotomy in the Westminster Hospital. Our simple Parisian *confrére* takes it all for Gospel, as dressed up in the English journal; and then makes the following reflection on English teachers and English *pupils*.

"Le journal Anglais blâme severement la conduite de ce medecin, et *des eleves*. Quel nom donner, en effet, à un oubli pareil des convenances? La *grossierité*, *l'ignorance*, la *cruauté*, ne saurient être portées plus loin. Nous sommes heureux de ne pas rencontrer de semblables scenes en France; elles dishonorent un medecin, et l'on ne peut que gemir d'etre obligé de les faire connaitre."

Such is the character for *coarseness*, *ignorance*, and *cruelty*, which is to meet the English medical man wherever he travels on the Continent! And why is France *so happy* as not to witness such *scenes* as the above? Because neither France, nor any portion of this globe, *except England*, is, or has ever been, *so unhappy* as to produce a monster capable of *inventing* them!

### 101. Vinum Colchici.

We have received several communications from chemists, complaining that they are in a dilemma respecting the mode of preparing this medicine, now become so important an article in practice. Thus Dr. Williams, in his work on Colchicum, directs two ounces of the dried seeds of colchicum to be infused, for eight or ten days, in a pint of sherry, and then to be strained. Dr. Thomson, in the last edition of his Dispensatory, orders ℥iss. of the dried bulb, or ℥j. of the dried petals or the *seed*, in ℥xij. of white wine. The College of Physicians directs *a pound* of the fresh root, four ounces of proof spirit, and eight ounces of distilled water—that is, a pound of solid to twelve ounces of menstruum! It is evident that this was meant for a poultice—for a wine or tincture it cannot be. Indeed, one of the first chemists in London publickly says the medicine cannot be made. At all events, it is clear that no two formulæ of this wine are of equal strength; and as physicians and surgeons do not make allusion to any particular formula, the chemist is left to make the preparation in any proportion that suits his taste. We have reason to believe that there are twenty different formulæ in this metropolis for the wine of colchicum!

It is curious that Dr. Thomson translates the formula of the London Pharmacopœia thus:

"Take of the fresh root of colchicum, bruised, *two ounces*; proof spirit, *twelve fluid ounces*; distilled water, *twenty fluid ounces*. Macerate for 14 days."

The formula in the Pharmacopœia runs thus:—

Rad. colch. recent. .. ℔j.
Spir. ten. ........ fl. ℥iv.
Aquæ distill. ......... ℥viij.

Thus the original directs a pound of the root to twelve ounces of fluid—the translation makes it *two ounces* of root to *thirty-two* ounces of menstruum!!

Such is the glorious uncertainty of physic—or at least of colchicum! We would recommend Dr. Williams' formula, when the seeds are used. In respect to the root, Dr. Thomson's formula appears as extravagantly weak, as that of the College is preposterously strong. Four ounces of the fresh root, or two ounces of the dry, to the pint of menstruum, would probably be a good standard.

As for the confusion among chemists and druggists, in consequence of the troy and avoirdupoise weights, it is beyond all description! It is fortunate that medicines admit of much latitude in their doses, and that many of them have little or no operation, otherwise the half the community would stand a chance of being poisoned by the mistakes that are daily made.

P.S. Since writing the above, we learn that the vinum colchici, as directed by the College, may be, and is made, by means of an hydraulic press, in the house of Messrs. Herring, brothers, of Aldersgate Street. They use wine instead of spirit and water, and, by means of the said press, they obtain one fourth more of the filtered and saturated wine than is originally employed. Thus, if 40 pounds of the fresh root of colchicum be infused in 40 pounds of wine, there will be 50 pounds of vinum colchici procured by the hydraulic press. We have no doubt that *this wine* is infinitely superior to the common v. colchici, procured in the usual way. It must contain the whole juice of the root. We learn that Messrs. Herring find the wine a better menstruum than the spirit and water, as it keeps. It is hardly necessary to say, that those who have not an hydraulic press, cannot make the vinum colchici according to the London Pharmacopœia.

---

102. GASTRO-ENTERALGIA. By M. LEGALLOIS.

Our readers will remember that we gave a full analysis of M. Barras' excellent work on "Gastralgia mistaken for Gastritis." This mistake is daily made on both sides of the Channel—especially since the doctrines of Broussais came to predominate. The following case, in high life, is not without parallels, we have reason to know, upon English soil.

*Case.* The COUNT DE C. aged 63 years, of bilious temperament, and subject, for several years, to a "hypochondriac gastro-enteralgia," had the imprudence to swallow, while in a state of perspiration after hunting, some cold drink. In a short time afterwards, he experienced very acute pain in the region of the duodenum, sense of constriction at the epigastrium, and yellow suffusion on the face. TWO HUNDRED LEECHES were applied in different relays, while warm baths, emollient lavements, sinapisms, and tartar-emetic ointment were employed on the surface. The pain ceased, but a state of great depression supervened. The skin was dry, the cheeks coloured, the muscles of the face were convulsed, the tongue was moist. The sense of constriction at the epigastrium was rather relieved than increased by pressure. The abdomen was contracted, the pulse small and quick, the intellectual faculties weakened, and the muscles of one arm tormented with spasms. At this period, Dr. Serrieres was called in, and found the region of the spleen very full and pulsating, and the colon loaded with indurated fæces. A purgative enema brought away large quantities of dark, fetid, and scybalous matters. Considerable relief followed; but at the next visit, Dr. S. found the patient in a state of stupor and delirium, with cool skin, and pulse scarcely perceptible. The case was now considered as most desperate. Three grains of James's powder were exhibited, and brought out a general perspiration, with elevation of the pulse. In a few hours afterwards, the dose of James's powder was doubled, and several black stools were evacuated. The symptoms were now greatly mitigated. The medicine was continued—a copious sediment was precipitated, and the disease took a critical turn. In a fortnight from the commencement of the attack, the patient was apparently in a state of convalescence. In two days after this favourable report, things took another turn. The sinapisms which had been discharging freely, were observed to become suddenly dry, followed by pain in the hypogastric region, dysury, scanty and red urine, sense of burning in the urethra. These symptoms were met by gentle means, and in ten days he was again convalescing. He was now allowed wine, and abused the indulgence, in consequence of which he had two relapses, in one of which he was nearly moribund, but was saved by bark, valerian, assafœtida, and stimulants. After various vacillations, ascites took place, and the water was obliged to be drawn off. When paracentesis was a second time deemed necessary, a copious flow of urine and perspiration produced resorption of the effused fluid. In three months from this time, the patient was restored to health.—REV. MED.

"What would have become of this patient, M. Legallois asks, if he had continued under the hands of a physiological physician?" The disease, from first to last, would doubtless have been considered as gastro-enteritis, and the patient would probably have perished under leeches and starvation. The ultra-depletion produced by two hundred leeches, for spasmodic pain in the stomach or bowels, resulting from cold drink, was most injudicious. A large dose of opium and camphor would have saved a great deal of the miseries which the poor Count was destined to undergo from the Sangrado practice first instituted.

---

### 103. MORBID ANATOMY OF THE LUNGS.

[Lectures attributed to Mr. Abernethy.]

In pursuing our criticisms on the doctrines and practices said to be inculcated by Mr. Abernethy, in his lectures, and long circulated among the profession without any contradiction of their accuracy, we again declare that we do not mean to tax the *lecturer* with any false doctrine or mala praxis, but merely to examine the *lectures* which are put forth in his name. Considering the length of time which has now elapsed since these lectures were first disseminated through the medium of the press, it certainly appears to us unaccountable that, out of so many thousand pupils as Mr. A. must have had in the last 20 or 30 years, not one should come forward to challenge or point out the discrepancies between the real and the spurious lectures! But to business.

The lecturer (always meaning the assumed lecturer) informs us that the lung is "one of the organs in which, when diseased action is produced, the disease is *tubercular*, as in the liver and spleen." Thus under the head of "morbid anatomy of the lungs," there is no notice taken of any of those structural changes which we daily see in the respiratory apparatus—except TUBERCULATION! After this, we cannot wonder at the following sentiment.

"This morbid anatomy, which people dread so much, *does not appear to me to be of primary importance in the study of our profession, there is such a great diversity of appearances;* but organs of certain construction are liable to certain diseases, and those are easily recognised; the grand thing to be attended to is, that what produces a state of irritation, and brings on a state of vascular action, will cause those diseases." 397.

These are the sentiments indeed which we have re-echoed in our medical societies. Morbid anatomy! Pooh! It is not worth a thought. The real reason for this contempt of pathology we shall presently shew.

"Now, with regard to the lungs, what can produce an irritable disease of pulmonary irritation? *I am very well convinced, that stomachic irritation will; and I am very well satisfied that this is the primary state of the disease.* I know that many gentlemen who have been educated at this hospital, have obtained very considerable credit to themselves, in curing or relieving some consumptive people by putting their stomachs to rights. I cannot tell you *all the cases* that I have known of this kind, but *one* I will tell you of, and this was the very first thing communicated to me, which produced those crazy opinions that have predominated in my mind ever since: there was a gentleman who attended these lectures, just about the time I first began to give them; he was a young Highlander, and a more intellectual or honourable man I never met with; he went to settle about five or six miles out of town, and both he and myself being young, I used to go to see him, and he used occasionally to come to see me. Once when I went to see him, he said, 'O, good God, what would I give for your opportunities! When we came first to this country, our books were crowded with consumptive cases, and I am convinced that consumption begins in the liver; I wish you would send me down some consumptive people; I will pay for their board, and attend them for nothing.' Now this being a clear-headed man, I listened to what he said, and I did send him down some patients; *but he could not cure them*, though he certainly did relieve them."

When we found that only one case was to be selected, out of the innumerable cures of consumption through the medium of the stomach, we did expect that *it* would be what Goldsmith would have called a "*thumper*." But, after all, it turns out to be merely the transfer of some consumptive patients from Mr. Abernethy in London, to the "intellectual highlander," six miles in the country—where, by the way, the patients were "not cured," but only "relieved"—a very convenient phrase long used in hospitals, when patients go out *nearly* as well as when they came in! Whether the relief in question resulted from the stomach-medicines prescribed by the "intellectual highlander," or from the *change of air* from town to country, we leave to the judgment of those who have made any observations on the effects of such changes.

The lecturer admits that, in some cases, there may be disease both in the lungs and stomach—"but then, what does that prove? it does not prove which of the organs is the most diseased." Certainly not. Nor does it prove the priority of disease in one organ rather than in another. The following passage affords a fine specimen of what may be called the *surgical pathology* of phthisis.

"But can consumption be cured? Odd bless me, that is a question which a man who had lived in a dissecting-room would laugh at: how many people do you examine who have lungs tubercular, but which are otherwise sound. What is consumption? It is tubercle of the lungs; then, if those tubercles were healed, and the lungs otherwise sound, the patients must get better. But if the enquirer shifts his ground, and says, it was the case I meant of tubercles over the whole of the lungs, why then he shifts his ground for no purpose; for there is no case which, when it has proceeded to a certain extent, can be cured. Therefore I say consumption may be cured, according to surgical principles, by creating local irritation on the side of the chest, and so on." 400.

Now the jet of the whole doctrine appears to be this:—stomach disorder is the primary state of disease in phthisis—ergo, cure the stomach-disorder, and you cure consumption. And as blue pill will cure stomach-disorder, why have recourse to any other medicine?—why trouble our heads about any other pathology, seeing that stomach-disorder is "the primary state of disease" of the lungs in pulmonary consumption? This reasoning is very plausible; and is very similar to that which was employed by the founder of our ancient and excellent ally the Turks. When Amru, the Mahomedan general, issued his mandate to burn the Alexandrian library, a deputation of literati waited on him, to dissuade him from his purpose. He asked them if the library contained any other books than the Koran; and being answered in the affirmative, he observed that, as the Koran contained every thing that was *necessary* to be known, so all books containing more than the Koran were *unnecessary*—and therefore he ordered the library to be burnt. The principles of Amru and Abernethy are precisely similar. Stomach-disorder is the cause of consumption—blue-pill and black-draught will cure stomach-disorder—ergo, all other pathology is erroneous—all other medicine is unnecessary.

---

### HOSPITAL PRACTICE.

### 104. HÔTEL DIEU.

As the taste of the day is decidedly in favour of hospital cases and hospital reports, we shall endeavour to make this department of the Journal as extensive as we can. There are several *velocipede* vehicles running in the same line of road with ourselves, but as the wares they carry are more exclusively of English growth, we shall generally prefer articles of foreign manufacture; in other words, we shall make it our business to give a full account of the practice of the French hospitals. Hospital reporting is of much more ancient date abroad, and
